# Quality and Safety of Meat Products

**DOI:** 10.3390/foods9060803

**Published:** 2020-06-18

**Authors:** Begoña Panea, Guillermo Ripoll

**Affiliations:** 1 Centro de Investigación y Tecnología Agroalimentaria de Aragón (CITA), Unidad de Producción y Sanidad Animal, Avda. Montañana 930, 50059 Zaragoza, Spain; gripoll@aragon.es; 2 Instituto Agroalimentario de Aragón–IA2 (CITA)-Universidad de Zaragoza, 50013 Zaragoza, Spain

**Keywords:** poultry, beef, lamb, carbon monoxide, volatile compounds, packaging, enhanced meat, sensory analysis, Campylobacter, *Escherichia coliO157:H7*, Hermetia illucens, Listeria monocytogenes, *Staphylococcus aureus*, Salmonella

## Abstract

Food safety is a major problem around the world, both regarding human suffering and with respect to economic costs. Scientific advances have increased our knowledge surrounding the nutritional characteristics of foods and their effects on health. This means that a large proportion of consumers are much more conscious with respect to what they eat and their demands for quality food. Food quality is a complex term that includes, in addition to safety, other intrinsic characteristics, such as appearance, color, texture and flavor, and also extrinsic characteristics, such as perception or involvement.

Food-borne diseases are a main problem in the meat industry [1]. The study of pathogens present in meat and meat products is crucial for the industry, for sanitary administration and to generate consumer trust. *Escherichia coli O157:H7* is one of the most important and studied pathogens present in fresh meat and it has been considered a contaminant of raw, non-intact beef products since 1999 [2]. A recent survey showed that 40–58% of US consumers ordered beefsteaks at medium rare (60–62.8 °C) to rare (54.4–57.2 °C), which could potentially put consumers at a high risk from *E. coli O157:H7* [3]. Some techniques should be implemented to reduce this risk. Li, et al. [4] conducted a study on coarse ground beef and veal patties, which aimed to investigate the quality variances, including color variation, during aerobic storage and cooking, as well as to evaluate the thermal inactivation of *E. coli O157:H7.* They hypothesize that a higher internal temperature with a longer rest time will increase the inactivation of *E. coli O157:H7* in beef and veal patties. The results showed that *Escherichia coli O157:H7* was more sensitive to heat in veal compared to beef, with shorter D-value. Cooking to 71.1 and 76 °C reduced *E. coli O157:H7* by >6 log CFU/g, regardless of rest time. Cooking to 55 °C and 62.5 °C with a 3.5 min rest achieved an additional 1–3 log CFU/g reduction compared to the 0.5 min rest. These results should be useful for developing a risk assessment of non-intact beef and veal products.

Behind this well-known pathogen, an additional problem is the emergence of other pathogens. Among this group of infectious bacteria, *Salmonella* spp., *Listeria monocytogenes*, *enterotoxigenic*, *Staphylococcus aureus* and *Campylobacter* spp. are the main contaminants in food due to their high occurrence worldwide and being major causes of gastroenteritis in humans [5,6]. Goncalves-Tenorio, et al. [7] published a meta-analysis, which summarized the levels of incidence of *Salmonella* spp., *Listeria monocytogenes*, *Staphylococcus aureus* and *Campylobacter* spp. in poultry meat commercialized in Europe. The results suggest that *S. aureus* is the main pathogen detected in poultry meat (38.5%; 95%CI: 25.4–53.4), followed by *Campylobacter* spp. (33.3%; 95% CI: 22.3–46.4%), while *L. monocytogenes* and *Salmonella* spp. present lower prevalence (19.3%; 95% CI: 14.4–25.3% and 7.10%; 95% CI: 4.60–10.8%, respectively). Despite the differences in prevalence, all of the pathogens were found in chicken and other poultry meats, at both the end-processing step and at retail level, in packed and unpacked products and in several meat cutting types. Prevalence data on cold preservation products also revealed that chilling and freezing can reduce the proliferation of pathogens but do not inactivate them.

Because cold application is not enough to prevent meat spoilage, it should be complemented with other technologies, such as packaging. During the last few decades, the modified atmosphere packaging (MAP) of foods has been a promising area of research, but much remains to be known regarding the use of unconventional gases, such carbon monoxide (CO). The use of CO for meat and seafood packaging is not allowed in most countries due to its potential toxic effect, and its use is controversial in some countries. Djenane and Roncalés [8] undertake a review to present the most comprehensive and current overview of the widely available, scattered information about the use of CO in the preservation of muscle foods. The conclusions shown that the use of CO in fresh meat packaging gives promising results due to its positive effects on overall meat quality, especially on color. The results stated that the risk of CO toxicity from the packaging process or from consumption of CO-treated meats is negligible and, additionally, CO is not present in the pack during storage. Some recommendations and future prospects addressed to food industries, consumers and regulators were pointed as “best practices”.

However, the packaging causes changes in meat quality, especially in color and flavor and, since these attributes are used by consumers to evaluate meat freshness, these changes due to the packaging must to be investigated [9]. A study was carried out by Karabagias [10] to evaluate the volatile profile of raw lamb meat during storage under refrigeration and to evaluate the aroma evolution of raw lamb packaged in a multi-layer coating film and stored at 4 ± 1 °C, as well as to investigate whether specific aldehyde ratios could serve as markers of lamb-meat freshness and degree of oxidation. Volatile compounds were determined using headspace solid phase microextraction coupled with gas chromatography/mass spectrometry. The results showed that volatile compound content increased during storage time and that the evolution of aldehydes during storage recorded a positive Pearson’s correlation (r) (*p* < 0.05) with the degree of oxidation (mg malonic dialdehyde per kg of lamb meat). In addition, a perfect Pearson’s correlation (r = 1) was obtained for the ratio hexanal to nonanal and, therefore, this ratio was proposed as an indicator of lamb meat freshness and overall quality.

Besides safety, another important issue for farmers and retailers is the cost-effectiveness. On the one hand, the main cost for farmers, especially those that reared monogastric animals, is feedstuffs, which in turn is dependent on soybean global markets and prices. Then, other protein sources have been investigated and algae and insects seem to be good substitutes. For this purpose, Altmann, et al. [11] raised 132 Ross 308 male birds on amino acid balanced diets, where 50% of the soy-based protein was substituted by either spirulina powder (*Arthrospira platensis*) or *Hermetia illucens* partially defatted larval meal, in starter and grower diets. Slaughterhouse parameters and meat physico-chemical and sensory properties were investigated. The results showed that meat quality could be improved when spirulina replaced 50% of the soy protein in broiler diets, although this substitution resulted in a dark red-yellow meat color. Besides, the substitution with *Hermetia* larval meal resulted in a product that did not differ from the standard fed control group, with the exception that the breast filet had a more intense flavor that decreased over storage time. Then, it was concluded that spirulina and *Hermetia* meal have the potential to replace soybean meal in broiler diets without deteriorating meat quality.

To modify feedstuff also allows us to obtain new products and, if these products are elaborated using the less valorized carcass joints, we have a feasible strategy to improve the incomes of farmers. This idea is the basis of the experiment carried out by Guerrero, et al. [12]. The paper assesses consumer acceptability of a cured product (“Cecina”) elaborated with cull ewes meat finished with different levels of linseed (5, 10 or 15%) for different periods before slaughtering (30, 50 or 70 days). The results show that linseed supplementation was identified as the most important factor for sensorial attributes (*p* < 0.01), with the preferred “Cecina” being that with 5% and 10% supplementation.

Another major threat to the sector is the constant decrease in meat consumption over the world [13]. The industry answered by implementing two main strategies: product enhancement and the development of new products.

Several approaches are possible to enhance a product [14,15]. A commonly used alternative is the addition of substances that improve the physical properties of the food (e.g., moisture enhancers). This technology improves tenderness, juiciness, flavor, and consistency of whole-muscle meat products, especially in those of reduced eating quality [16,17]. Unfortunately, with the current industry for brine injection, brine is not uniformly distributed throughout the meat. Vacuum impregnation allows for the direct insertion of an external solution into a product through its pores in a fast, controlled and uniform way without destroying its original structure [18]. Leal-Ramos, et al. [19] conducted an experiment to vary the pressure, (20.3, 71.1 kPa) and time, (0.5, 2.0, 4.0 h) of impregnation. The results showed that both the vacuum and atmospheric pressures generated a positive impregnation and deformation. The highest values of impregnation (10.5%) and deformation (9.3%) were obtained at a pressure of 71.1 kPa and a time of 4.0 h. The sample effective porosity exhibited a significant interaction (*p* < 0.01) between pressure and time and the highest value (14.0%) was achieved at a pressure of 20.3 kPa and a time of 4.0 h, whereas the most extended distension of meat fibers (98 μm) was observed at the highest levels of pressure and time. These results indicate that meat from mature cows can undergo a vacuum-wetting process successfully, with an IS of sodium chloride to improve its quality.

The enhancement can also be afforded regarding nutritional value and, in this approach, the search for low-calorie products has been widely studied and developed in several kind of products [20]. Bechtel, et al. [21] aimed to make a battered catfish product that could be baked with a lower percentage of oil-based calories than the equivalent par fried products. Then, the effect of different batters (rice, corn, and wheat) was examined and the effect of par frying on the composition and texture properties of baked catfish. The results found that the lipid contents of the par fried treatments were significantly higher for both corn and wheat batters than for non-par fried treatments. Sensory analysis indicated that the texture of the coatings in the par fried treatments were significantly greater for hardness attributes. In addition, fillet flakiness was significantly greater in the par fried treatments and the corn-based batters had moister fillet strips compared to the wheat flour batters.

Finally, interest in convenience products has increased in the last few years because the patterns of food and meat consumption are constantly changing, not only due to socioeconomic and cultural trends that affect the whole society, but also to the specific lifestyles of consumer groups [22]. Ripoll, et al. [23] carried out a study to identify the profiles of lamb meat consumers according to their orientation toward convenience, also analyzing their socioeconomic characteristics and their preferences regarding the intrinsic and extrinsic quality signals of lamb meat and their willingness to pay for lamb confit. Four types of consumers were differentiated according to their lifestyles related to lamb consumption: “Gourmet”, “Disinterested”, “Conservative” and “Basic”. The Gourmet group has characteristics that make it especially interesting to market a product, such as lamb confit, and consequently, marketing strategy should to be focused to this niche market.
